# The effect of manual therapy on pulmonary function in healthy adults

**DOI:** 10.1038/srep33244

**Published:** 2016-09-12

**Authors:** Bradley A. Wall, Jeremiah J. Peiffer, Barrett Losco, Jeffrey J. Hebert

**Affiliations:** 1 School of Psychology and Exercise Science, Murdoch University, Murdoch, 6150, Australia; 2 School of Health Professions, Murdoch University, Murdoch, 6150, Australia; 3 Faculty of Kinesiology, University of New Brunswick, Fredericton, E3B 5A3, Canada

## Abstract

Manual therapy is suggested as a potentially therapeutic intervention that may improve pulmonary function. However, this form of therapy is largely based on clinical observations and hypothetical models rather than mechanistic knowledge. This study examined the influence of a single session of manual therapy applied to the thoracic spine and thorax on dynamic pulmonary function over an extended time frame in healthy adults. 21 healthy individuals (14 males) aged 19–35 (mean [SD] age = 23 [3.9], BMI [SD] = 22.97 [2.41]) completed one experimental testing session consisting of five pulmonary function tests and the delivery of a manual therapy intervention. Pulmonary function was measured at baseline and 1 minute, 10 minutes, 20 minutes and 30 minutes following the intervention. Baseline mean (SD) forced vital capacity (FVC), forced expired volume in 1 second (FEV_1_) and maximal voluntary ventilation (MVV) were 5.55(1.23 L), 4.64(0.92 L) and 165.7(40.0L min^−1^) respectively. The mean (SD) FEV_1_/FVC ratio was 0.84(0.07). There were no statistically significant changes in any of the pulmonary function measures following the manual therapy intervention. Our findings do not support the use of manual therapy to provide a short-term benefit in respiratory function to healthy adults.

Thoracic manual therapy is a widely used manipulative technique in clinical practice. However, this form of therapy is largely based on clinical observations and hypothetical models rather than mechanistic knowledge^1^. Specifically, research investigating the physiological outcomes of this form of therapy in healthy adults is currently lacking. Manual therapy to the spinal region is proposed to increase joint mobility^2^ which could exert a positive influence on chest wall compliance and pulmonary function, a theory which has been previously investigated in those with respiratory system limitations such as chronic obstructive pulmonary disease and asthma^3^,^4^,^5^,^6^. Thoracic manual therapy has been suggested as a therapeutic intervention with the potential to improve respiratory function among healthy individuals^7^, however research supporting this claim is limited.

Previous thoracic manual therapy studies have investigated sympathetic nervous system activity and have reported increased in respiratory rate, heart rate, and blood pressure following thoracic manipulation^1^,^8^. With the majority of the literature using patients with existing respiratory system limitations^3^,^5^, very few studies have looked at the impact thoracic manual therapy may have on respiratory function in healthy adults^7^. One such study has reported improved respiratory function following thoracic manual therapy, however, is constrained by several factors. First, the sample of participants comprised individuals categorized as being in the lower end of healthy lung function. Additionally, the improvements in respiratory function occurred following a series of six manual therapy treatment sessions; thus may not accurately reflect the use of thoracic manual therapy in an acute clinical setting. Finally, the effects of thoracic manual therapy on respiratory function have only been measured in the period immediately following treatment^3^,^7^ and therefore the duration of these potential effects remains unknown. The duration of the treatment effect of thoracic manual therapy on respiratory function can have practical implications regarding potential enhancement of respiratory muscle function in healthy individuals. Therefore, this study examined the influence of a single session of thoracic manual therapy applied to the thoracic spine and thorax on dynamic pulmonary function over an extended time frame in healthy adults. To date, no previous studies have looked at the time course of any potential physiological benefits elicited by spinal manipulative therapy.

## Methods

A pre-test, post-test single-arm trial involving repeated measures in the post-intervention period was performed. The trial was prospectively registered with The Australian New Zealand Clinical Trials Registry (ID: ACTRN12612001141831 Date: 7 November 2012). The trial protocol received ethical approval from the Murdoch University Human Research Ethics Committee and all methods were performed in accordance with the Committee’s guidelines and regulations. All participants provided, in writing, their informed consent to participate prior to study enrolment^9^.

### Participants

Twenty participants (14 males) were recruited (mean [SD] age = 23 [3.9], BMI [SD] = 22.97 [2.41]) from a university campus to participate in this study. Potential participants were excluded if they reported; 1) any history of pulmonary disease, 2) smoking within the previous 12 months, 3) use of medications known to influence pulmonary function (e.g asthma medication), 4) pregnancy or 5) contraindications to manual therapy.

### Pulmonary function

All participants completed one experimental testing session which involved the delivery of a manual therapy intervention as well as pulmonary testing at five-time points. Pulmonary function was measured at baseline and 1 minute, 10 minutes, 20 minutes and 30 minutes following the thoracic manual therapy intervention. Upon arriving at the Murdoch University laboratory, participants completed a baseline pulmonary functional test (PFT) and a test of maximal voluntary ventilation (MVV). The PFT comprised forced vital capacity (FVC) and forced expiratory volume in 1 second (FEV_1_) measurements which are representative of the volume of air that can be forcibly expelled following full inspiration and used in the diagnosis of obstructive and pulmonary diseases. MVV is the maximal ventilation achievable during 15 seconds of voluntary hyperventilation. All PFT and MVV measures were completed in a seated position using a hand-held spirometer connected to a ParvoTrueOne metabolic cart (ParvoMedic, USA). Before testing, the metabolic cart was calibrated, as per manufacturer’s specifications, through a range of inspiratory and expiratory flow rates using a 3-L syringe (Hans Rudolph, USA). During the PFT, participants were instructed to breathe normally into the spirometer for 30 seconds after which they were instructed to forcefully inspire maximally and then maximally expire as forceful as possible for six seconds. All PFT measures were completed in triplicate, allowing one minute between efforts, with the best results used for analysis. From the PFT, forced vital capacity (FVC), forced expired ventilation in one second (FEV_1_) and the FEV_1_·FVC^-1^ ratio was calculated. Sixty seconds after completing the third PFT, participants were required to complete a single MVV maneuver. During this test, participants were instructed to breathe normally into the spirometer for 30 seconds after which they inhaled and exhaled as deep and fast as possible for 15 seconds. Ventilation data for the best continuous 12-second epoch was determined via the ParvoTrueOne software and used to estimate the 1-minute MVV.

### Thoracic manual therapy treatment

The thoracic manual therapy intervention involved a single treatment session comprising two manual therapy techniques applied in standardized fashion: 1) high-velocity, low-amplitude thrust manipulation and 2) low-velocity joint mobilization. The thrust manipulations were targeted at the upper (T1-T3), mid (T4-T7), and lower (T8-T12) thoracic spine. The high-velocity techniques involved thrust manipulations with the participant in the seated or supine positions, depending on the clinician’s judgement and the preference of the participant. If a joint cavitation was experienced with the thrust manipulation, the clinician proceeded to the next region. If no joint cavitation was experienced, the participant was repositioned and the technique was repeated. When no joint cavitation was experienced after the second attempt, the clinician proceeded to the next spinal region or technique. Additional details of these manipulation techniques have been reported previously. 10,11 Following the thrust manipulations, the treating clinician performed low-velocity, rotatory, grade 4 joint mobilizations to the thoracic spine and/or costovertebral joints. With the participant seated and with hands placed on the contralateral shoulder, the clinician placed their hand on the spine or costovertebral joint and rotated the participant toward end-range. Each participant received one to three sets of 10 mobilizations to the left and right. The number of low-velocity mobilizations was determined by the clinician’s discretion. Similarly, mobilizations were directed at joints and regions perceived by the clinician to be most stiff. Further details of the low-velocity mobilization technique are available elsewhere.^12^ Total treatment time was approximately 5 minutes. A clinician with twelve years of clinical and manual therapy experience performed all treatments.

### Statistical Analysis

The minimum level of clinically important change in FVC and FEV_1_ is estimated to be 12%.^13^ Based on previous research,^14^ we assumed the baseline mean (standard deviation) FEV_1_ value would be 4.48 (0.6) literswith a correlation between measures of 0.3. Considering an alpha level of 0.05, recruiting 20 participants would provide 90% power to detect a 12% change in FEV_1_. Data management and statistical analyses were performed using the Statistical Package for the Social Sciences version 21.0 (SPSS, Inc., Chicago, IL). Treatment effects were estimated using separate, one-way repeated measures analysis of variance models for each of the three dependent variables. The independent variable was time with five levels (pre-treatment baseline, and measures at 1 minute, 10 minutes, 20 minutes and 30 minutes post-treatment) and the dependent variables were FVC, FEV_1_, and MVV. Data was assessed for normality using the Kolmogorov-Smirnov test. Alpha level was 0.05 for all analyses.

## Results

Baseline mean (SD) FVC, FEV1 and MVV were 5.55 (1.23 L), 4.64 (0.92 L) and 165.7 (40.0 L.min^−1^) respectively. The mean (SD) FEV_1_/FVC ratio was 0.84 (0.07). There were no statistically significant changes in the pulmonary function measures at any time point following the manual therapy intervention (Table 1). There were no adverse events reported.

## Discussion

This study examined the effect of a single session of manual therapy applied to the thoracic spine and thorax on pulmonary function in healthy individuals. The main finding from this study was thoracic manual therapy did not alter pulmonary function immediately following or up to 30 minutes after a thoracic manual therapy intervention.

While thoracic manual therapy is a commonly used treatment modality, very little research has investigated its effect on pulmonary function. It has been previously proposed that manipulative therapy may enhance joint mobility and subsequently, enhance static and or dynamic lung function^7^. This was not the case in the present study with our findings indicating that a single thoracic manual therapy treatment applied to the thoracic spine and thorax does not result in immediate or transient (30 minutes) changes in pulmonary function. These findings are in contrast to Engel and Vemulpad^7^ who reported increases in FVC/FEV_1_ lung function measures following nonspecific high-velocity low-amplitude manipulation of the lower cervical and thoracic spine, and the posterior articulations of the associated ribs in healthy individuals. Importantly, these findings were only reported immediately following the sixth manual therapy session during an intervention consisting of six sessions over a four week period^7^. Further, the sample of participants in that study comprised individuals with low FVC readings (20 of the original 41 participants) and thus does not appropriately represent the general population. In addition, we feel our findings represent a clinical scenario where an individual is likely to encounter with a single manipulative treatment.

The intervention in the current study consisted of only one manual therapy treatment. It is possible that additional treatments may favourably impact pulmonary function; however, the intervention applied in this current study is likely to be reflective of what can typically be done in a clinical environment. It is possible that the specific manual therapy treatment applied may have differed in application to that of previous research, which may partly explain the discrepancies in findings. A further limitation is the effect of the thoracic manual therapy treatment was only measured up to 30 minutes post treatment; and the potential for longer term effect on pulmonary function is unknown. However it is unlikely that pulmonary function would change after this period of time, especially considering that thoracic manual therapy and specifically high-velocity manipulation may be driven by changes in skeletal muscle function and joint stiffness^15^,^16^.

Research investigating the effects of thoracic manual therapy on pulmonary function in both healthy and impaired populations is currently lacking. While the current study results do not support the use of thoracic manual therapy for enhancing pulmonary function in healthy adults, alternative thoracic manual therapy approaches may yield different results. Therefore, future research efforts in this area should examine the effects of different manual therapy techniques and treatment protocols.

## Conclusion

Our findings do not support the use of thoracic manual therapy to provide a short-term benefit in respiratory function to healthy adults. As such, this treatment modality is unlikely to provide a benefit to already healthy individuals through further enhancements in respiratory function. Whilst no difference was seen at rest in healthy individuals, additional research is necessary to explore the possible effects of thoracic manual therapy in the context of enhancing performance in aerobic based sports where the respiratory system is considered a rate limited factor.

## Additional Information

**How to cite this article**: Wall, B. A. *et al*. The effect of manual therapy on pulmonary function in healthy adults. *Sci. Rep.* **6**, 33244; doi: 10.1038/srep33244 (2016).

## Figures and Tables

**Table 1:** Pulmonary function variables before and after thoracic manual therapy intervention.

	Mean (SD)	Mean difference from baseline (95% CI)
FVC (L)		
Baseline	5.55 (1.23)	—
1 minute	5.58 (1.16)	−0.03 (−0.17, 0.11)
10 minutes	5.56 (1.18)	−0.01 (−0.13, 0.11)
20 minutes	5.52 (1.14)	0.29 (−0.15, 0.21)
30 minutes	5.60 (1.20)	−0.05 (−0.20, 0.10)
FEV_1_ (L)		
Baseline	4.64 (0.92)	—
1 minute	4.61 (0.89)	0.03 (−0.69, 0.12)
10 minutes	4.54 (0.96)	0.10 (−0.21, 0.21)
20 minutes	4.57 (0.80)	0.07 (−0.07, 0.21)
30 minutes	4.63 (0.93)	0.01 (−0.10, 0.11)
MVV (L.min^−1^)		
Baseline	165.7 (40.0)	—
1 minute	164.3 (39.5))	1.3 (−4.3, 7.0)
10 minutes	165.0 (40.1	0.7 (−8.3, 9.6)
20 minutes	160.1 (40.7)	5.5 (−5.8, 16.9)
30 minutes	161.0 (37.6)	4.7 (−5.7, 15.1)

FVC, FEV_1_, MVV.
